# Enzymeless electrochemical detection of hydrogen peroxide using NiO octahedron decorated 3D graphene hydrogel

**DOI:** 10.1038/s41598-025-10472-6

**Published:** 2025-08-03

**Authors:** Mohamed A. Yassin, Ayman F. Abou-Hadid, Hamouda M. Mousa, Chan Hee Park, Cheol Sang Kim, Ali Salem, Mohamed A. Mattar

**Affiliations:** 1 Department of Biosystems Engineering, Institute of Postgraduate Studies and Agricultural Research in Arid Regions, Ain Shams University, Cairo, 11241 Egypt; 2 Horticulture Department, Faculty of Agriculture, Ain Shams University, Cairo, 11566 Egypt; 3 Department of Mechanical Engineering, Faculty of Engineering, South Valley University, Qena, 83523 Egypt; 4 Department of Bionanosystem Engineering, Graduate School, Jeonbuk National University, Jeonju, 54896 Republic of Korea; 5 Division of Mechanical Design Engineering, Jeonbuk National University, Jeonju, 54896 Republic of Korea; 6 Civil Engineering Department, Faculty of Engineering, Minia University, Minia, 61111 Egypt; 7 Structural Diagnostics and Analysis Research Group, Faculty of Engineering and Information Technology, University of Pécs, Pécs, 7622 Hungary; 8 Prince Sultan Bin Abdulaziz International Prize for Water Chair, Prince Sultan Institute for Environmental, Water and Desert Research, King Saud University, P.O. Box 2454, Riyadh, 11451 Saudi Arabia

**Keywords:** Self-assembly, Graphene hydrogel, Octahedrons, Electrochemical biosensors, Electrochemistry, Materials chemistry

## Abstract

Nowadays, electrochemical biosensors have gained extensive consensus to detect a wide variety of analytes such as hydrogen peroxide (H_2_O_2_), glucose, dopamine, uric acid, and so on. However, the detection of H_2_O_2_ is more and more important because H_2_O_2_ plays a vital role in our daily life. Hence, a new nonenzymatic H_2_O_2_ biosensor was developed by decorating NiO octahedrons on the 3-dimensional graphene hydrogel (3DGH). The NiO octahedrons were prepared using a mesoporous silica SBA-15 as a hard template. Then, different amounts of NiO octahedrons self-assembled with 3DGH during hydrothermal method. The performance of different prepared nanostructures as H_2_O_2_ biosensors electrodes was determined by the cyclic voltammetry and chronoamperometry tests. Various physicochemical methods such as FE-SEM, HR-TEM, XRD, TGA and Raman were utilized to characterize the morphology and structure properties of the as-prepared samples. The electrochemical sensing of H_2_O_2_ for the proposed biosensor electrodes are investigated, and the 3DGH/NiO nanocomposite electrode with NiO content of 25% displays high sensitivity with wide linear range and low detection limit, as well as good selectivity, reproducibility and long-term stability. Finally, the analytical utilities of the 3DGH/NiO25 proposed electrode were conducted to detect H_2_O_2_ in real products of milk samples.

**Supplementary Information:**

The online version contains supplementary material available at 10.1038/s41598-025-10472-6.

## Introduction

**Fig. 1:**
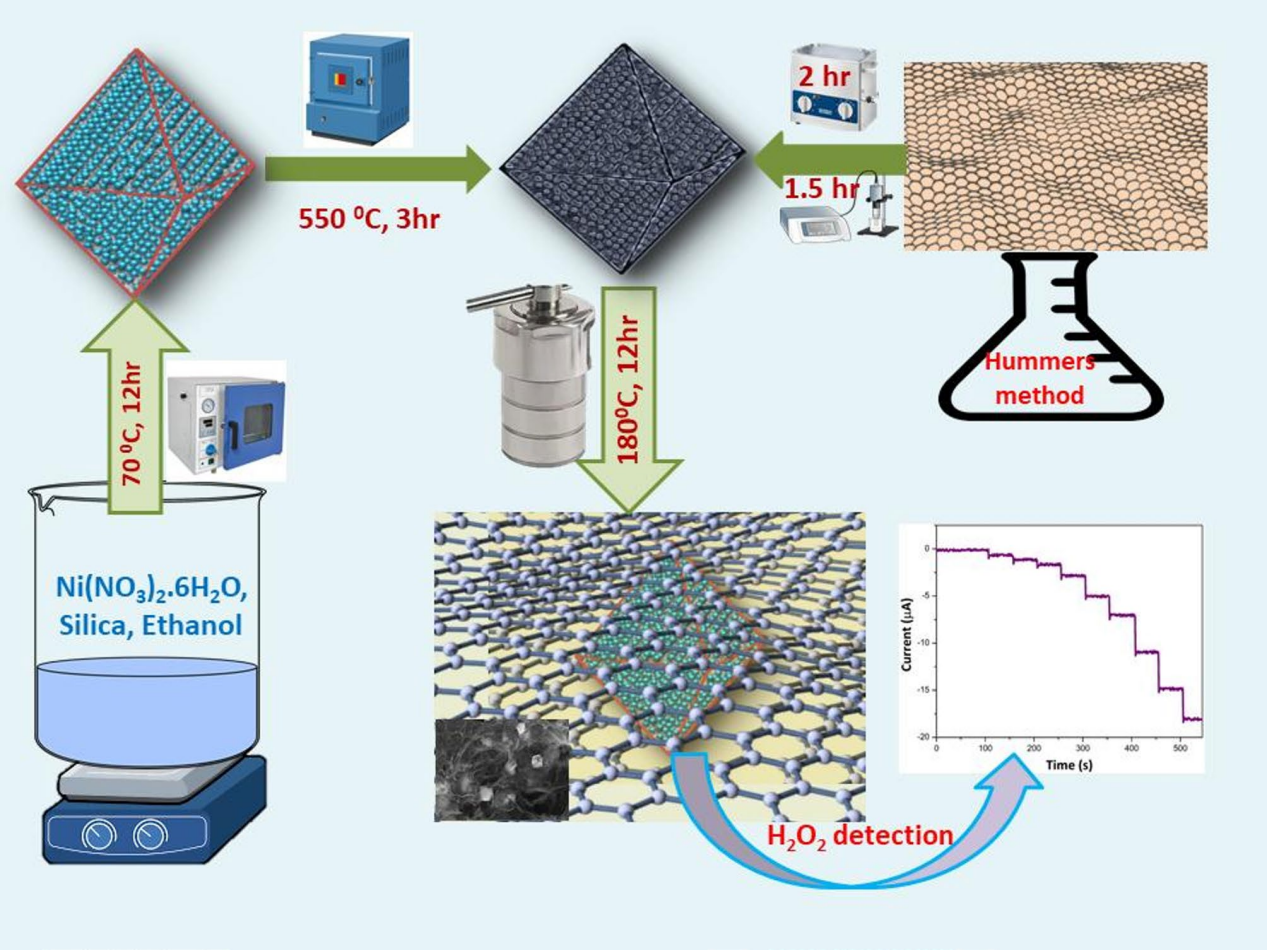
Schematic shows the fabrication of 3DGH/NiO nanostructures. The 3D visualization was created using Blender software (version 3.6, https://www.blender.org/).

In recent years, electrochemical biosensors (EBS) have garnered significant attention within the scientific community and industry, emerging as a pivotal technology in various analytical fields. This surge in interest is primarily attributed to their remarkable combination of cost-effectiveness, operational simplicity, and high sensitivity, which collectively address many of the challenges faced by conventional analytical methods^1–4^. The growing necessity for precise and efficient detection of a diverse range of analytes, including but not limited to Hydrogen Peroxide, Glucose, Dopamine, Ascorbic acid, and Uric acid, underscores the escalating significance of Electrochemical Biosensors (EBS) in both current and future analytical landscapes. This rising demand, coupled with the critical role these analytes play in maintaining human health and environmental safety, strongly suggests that EBS will assume an even more pivotal position in forthcoming scientific and technological advancements. Among these analytes, Hydrogen Peroxide (H_2_O_2_) plays an extremely important role in wide range of different processes such as a water disinfectant to replace chlorine or chlorine dioxide, food manufacturing, mining industry, bleaching of wood pulp and many other processes^5–8^. In addition, H_2_O_2_ has direct effect on our health due to the high content of H_2_O_2_ accumulation in cells would afford severe health risk of cell damage, including Alzheimer’s disease, cardiovascular disease, cancer and neurodegeneration disease etc^9–12^. Although enzyme-based biosensors have been investigated by many preceding efforts, the practical drawbacks including high cost, complicated fabrication and lack of stability could be restricted its commercial application^13–15^. Considering the critical importance of hydrogen peroxide (H_2_O_2_) detection in various fields, there is an urgent need to develop advanced materials with exceptional electrocatalytic properties. These materials are crucial for enhancing the sensitivity and stability of nonenzymatic H_2_O_2_ electrochemical biosensors, thereby improving their overall performance and reliability. In the pursuit of developing high-performance materials, transition metal oxides (TMOs) have emerged as particularly promising candidates. TMOs such as NiO, MnO_2_, Co_3_O_4_, and Fe_2_O_3_, among others, have garnered significant attention from researchers and industry experts over the past decade. This surge in interest can be attributed to their unique and advantageous properties, which make them exceptionally well-suited for a wide range of applications, with a particular emphasis on electrochemical detection^16–19^. One of the most common transition metal oxides is Nickle oxide (NiO). It has an excellent applied performance in many applications including lithium and sodium ion batteries^20–22^, supercapacitors^23–25^, gas sensors^26^ and solar cells^27^ due to its low toxicity, facile preparation, natural abundance and good electrochemical activities. More recently, different morphologies of NiO have received a lot of attention as electrochemical biosensors materials for detection of various analytes. For example, Fang et al. have modified MWCNT with nickel hexacyanoferrate nanoparticles for amperometric determination of uric acid^28^. Using graphene oxide film as template, Zhang and Liu fabricated NiO nanosheets for detection of glucose sensing^29^. Also, NiO nanofibers were prepared via electrospinning technique to modify glassy carbon electrode containing graphene oxide for improving performance nonenzymatic glucose biosensor^30^. Therefore, most literature confirmed that the integration among metal oxide and carbonaceous materials could greatly improve the performance of electrochemical biosensors by reinforcing the electron transport, ion diffusion and accessibility. Among of carbonaceous materials which have been used as supported materials, graphene has drawn huge attention due to its marvelous electronic and mechanical characteristics^31–33^. However, 2D graphene suffer from agglomeration and restacking from the strong interlayer interactions that can reduce the specific surface area and the number of electrochemically active sites^34,35^. Thus, 3D graphene hydrogel has attracted great attention recently in different analytical electrochemistry field, owing to its excellent physicochemical properties including large surface area, high intrinsic electrical conductivity, superior controllable pore size distribution and porosity^36–40^. For instance, Chen et al. prepared 3D porous prussian blue in graphene aerogels by reduction of graphene oxide and FeCl_3_ for detection of H_2_O_2_ sensor with a linear range (0.005–4 mM). In this case, L-ascorbic acid and ferricyanide were selected as a reducing agent and prussian blue source, respectively^41^. Yin and his coworkers synthesized Ni_3_N nanoparticles/3D graphene aerogel using hydrothermal reaction and then calcining under NH_3_ atmosphere. The composite was used for detection of glucose and H_2_O_2_ molecules^42^. Recently, Elsayed et al. introduced a biosensor for COVID-19 detection, combining graphene metasurfaces with gold, silver, and GST materials, optimized through COMSOL Multiphysics simulation in the infrared regime for high sensitivity and precision^43^. Hence, numerous previous studies demonstrated that metal oxides, hydroxides and nitrides decorated graphene could greatly enhance the electrochemical performance of various electrochemical biosensor applications. Inspired by the good properties of nickel oxide as an efficient electrocatalyst and graphene as supported materials, we have successfully prepared exemplary 3DGH/NiO octahedrons for nonenzymatic H_2_O_2_ detection applications. To our knowledge, there is a notable gap in the existing literature regarding the utilization of 3D graphene hydrogel as a sensing electrode material for the detection of H_2_O_2_. This presents an exciting opportunity for novel research in the field of biosensing technology. Considering this, our study aims to address this gap and explore the potential of graphene-based materials for H_2_O_2_ detection (Fig. 1). As a first step in our innovative approach, we focused on the synthesis of NiO octahedrons using nickel nitrate hexahydrate as nitrate source, ethanol as solvent and a mesoporous silica SBA-15 as a hard template. Second, different amount of NiO octahedrons ranged from 5 to 35% wt were decorated 3DGH via hydrothermal method. X-ray diffraction (XRD), thermogravimetric Analysis (TGA), field emission scanning electron microscopy (FE-SEM), high resolution transmission electron microscope (HR-TEM), Raman spectroscopy and electrochemical tests were applied to characterize the structural, morphological and electrochemical properties of the as-prepared samples. As a result, the optimized 3DGH/NiO25 nanocomposite exhibited a superior electrochemical performance toward H_2_O_2_ sensing with a good sensitivity (117.26 µA mM^−1^ cm^−2^), wide linear range (10 µM–33.58 mM) and low detection limit (5.3 µM).

## Materials and methods

### Chemicals and reagents

Graphite powder used for the synthesis of graphene oxide (GO) was purchased from Daejung Chemical Reagent Co (South Korea). Silicon dioxide (SiO_2_), Nickel (II) nitrate hexahydrate (Ni(NO_3_)_2_.6H_2_O) and sodium hydroxide pellets (NaOH) were obtained from Sigma-Aldrich Chemical Co. (USA). D-Glucose, Uric acid (UA), Dopamine hydrochloride (DA) and L-ascorbic acid (AA) were purchased from Tokyo chemical industry Co. Ltd from Bioshop Canada Inc. Ethyl alcohol (EtOH), H_2_O_2_ (38.5 wt %), Urea, NaNO_2_, NaCl, Na_2_HPO_4_, KH_2_PO_4_ and KCl were purchased from Samchun Pure Co. Ltd (South Korea). All reagents purchased are of analytical grade and used as received without any further purification. Phosphate buffer solution (PBS, 0.1 M, pH 7.4) was freshly prepared in ultrapure water purified using a Millipore-Q system.

### Preparation of nickel oxide (NiO) octahedrons

In a typical procedure, 10 mg of silica were dissolved into 100 ml of anhydrous ethanol (EtOH) containing 10 mg of nickel nitrate hexahydrate (Ni(NO_3_)_2_.6H_2_O) and stirred for 24 h at room temperature (RT). Then, the mixed solution was dried at 80 °C for 48 h. The powder was ground well and rinsed following the same procedure one more time. Subsequently, the dry product was transferred to a muffle furnace and calcinated at 550 °C for 3 h at a heating rate of 2 °C min^−1^. To remove the silica template, the final product was treated two times with 2 M NaOH at 60 °C and washed with ethanol and water repeatedly, and then dried in a vacuum oven at 70 °C for 12 h.

### Self-assembly of the 3D graphene hydrogel/nickel oxide octahedrons

First, 48 mg of graphene oxide (GO), which was synthesized by the oxidation of graphite powder according to a modified Hummers method as described previously,^44,45^ was dispersed in 32 mL of deionized water containing 12 mg of NiO octahedrons using bath-sonicated for 2 h and followed by prop-sonication for 1.5 h. After that, the mixture was transferred to a 45 mL Teflon-lined autoclave, and kept at 180 °C for 12 h. After natural cooling to room temperature, the product named as 3DGH/NiO25 was then washed numerous times by deionized water and finally dried by freeze drying. The identical procedure has been repeated for the formation of 3DGH/NiO5 (2.5 mg of NiO), 3DGH/NiO15 (7.5 mg of NiO) and 3DGH/NiO35 (17 mg of NiO).

### Spectroscopic characterization

The morphology of obtained as-fabricated samples was investigated through field emission scanning electron microscopy (FE-SEM, Carl Zeiss SUPRA 40VP, Germany) and high-resolution transmission electron microscopy (HR-TEM, H-7650 Hitachi Ltd, Japan). For investigating the crystallinity and phase structure of the samples, X-ray diffraction (XRD) observations were recorded using X-ray diffractometer (Rigaku, Japan) with high-intensity monochromatic Cu-Kα radiation as an incident beam (λ = 1.54 Å) over a Bragg’s angle range from 10° to 90°. Thermal stability and weight ratio of the prepared samples was analyzed using Q50 TGA device (TA instruments, USA). In addition, Raman spectroscopy measurements were recorded by a Nanofinder 30 (Tokyo Instruments Co., Japan).

### Electrochemical characterization

Electrochemical measurements were performed on a ZIVE SP1 electrochemical workstation (WonATech Co. Ltd. Seoul, Korea) with a three-electrode system, which consisted of the modified glassy carbon electrode (GCE) with a geometric surface area of 0.0707 cm^2^ (3 mm diameter) as working electrode, silver/silver chloride (Ag/AgCl, saturated KCl) as the reference electrode and platinum (Pt) wire as the counter electrode. The modified working electrode was fabricated by dispersion 2 mg of as-synthesized material into 400 µl of isopropanol in the presence of 20 μL of Nafion solution (5 wt %) under sonication condition to obtain homogeneous solution. Then, 15 µl of the prepared sample was coated on a bare glassy carbon electrode. Finally, the fabricated electrode was dried at 60 °C for 30 min. Electrochemical impedance spectroscopy (EIS) was carried out in 5.0 mM K_3_Fe[CN]^6^ as redox probe in 0.1 M KCl aqueous solution. However, cyclic voltammetry and chronoamperometry measurements were carried out in 10 mL of 0.1 M PBS (pH 7.4) solution without or with presence hydrogen peroxide and purged with high purity nitrogen for 10 min prior to each measurement. All electrochemical experiments were conducted at room temperature (25 ± 2 °C).

## Results and discussion

### Physicochemical characterization

**Fig. 2:**
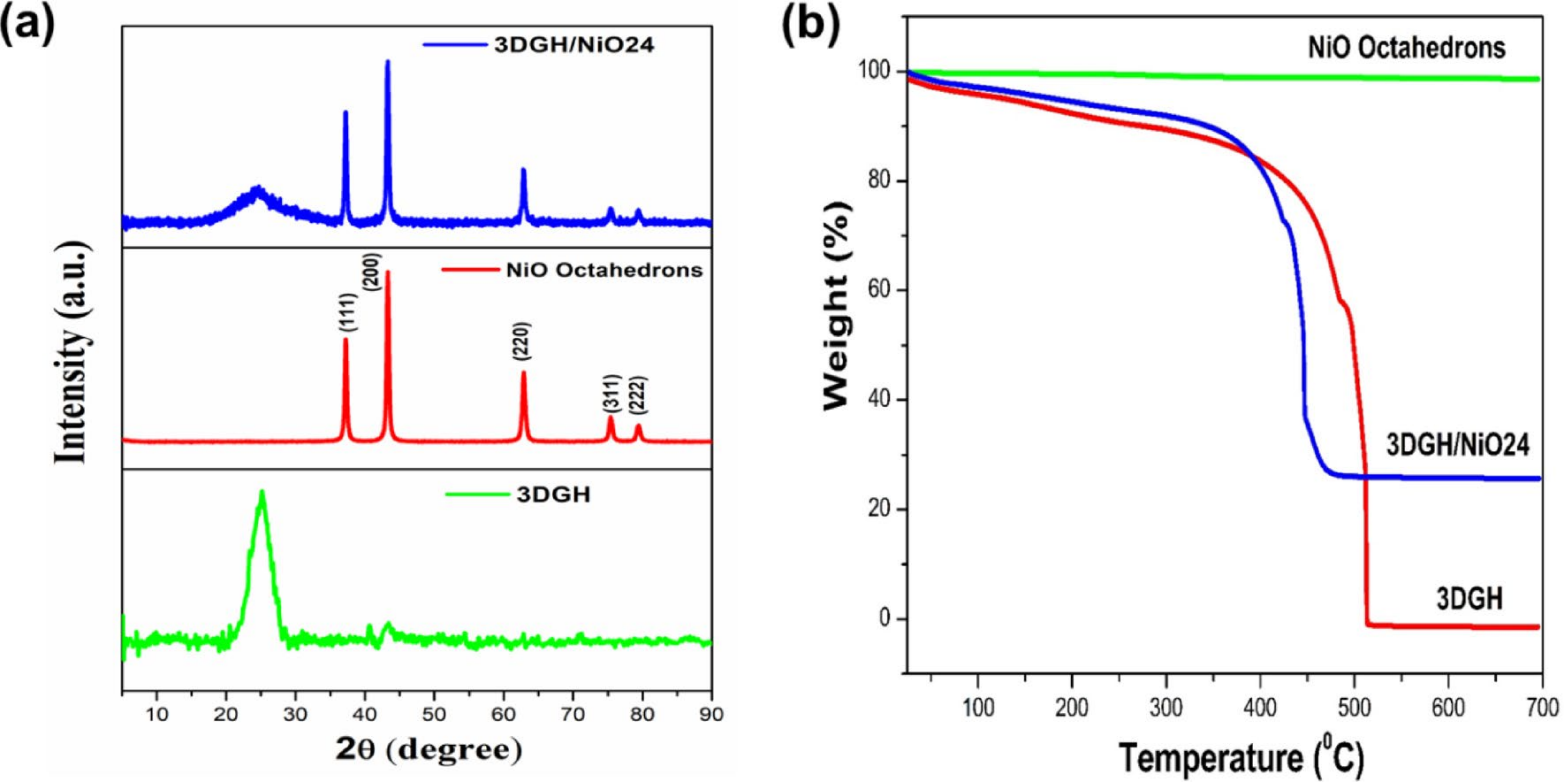
(**a**) XRD patterns and (**b**) TGA curves of 3DGH, NiO octahedrons and 3DGH/NiO25.

The crystal structure and the composition of the as-prepared samples were carried out by X-ray diffraction technique. As most clearly shown in Fig. 2a, an obvious single peak at 2θ = 25° (002) was observed for 3DGH, attributed to a few layers of obtained graphene hydrogel with fewer residual oxygen containing groups ^46^. In addition, the obtained XRD spectra has NiO main peaks at 2θ = 37.4°, 43.6°, 62.9°, 75.4°and 79.7° can be perfectly assigned to the (111), (200), (220), (311) and (222) crystal planes of the face centered cubic (FCC) NiO, respectively, which is in exact agreement with the (47–1049) standard card from JCPDS^47,48^ (Figure S2). Furthermore, different sharp XRD diffraction peaks of NiO can be observed in 3DGH/NiO25 nanocomposite, indicating the good synergistic of 3DGH and NiO which additionally confirms the NiO maintain its initial crystal structure during fabrication of 3DGH via hydrothermal process (Table S1). To get the properties of thermal stability and weight ratio of 3DGH, NiO and 3DGH/NiO25 nanostructures, thermogravimetric analysis (TGA) was conducted at air environment with a heating rate of 10 °C/min in the temperature range of 25–700 °C, as can be found in Fig. 2b. Evidently, it is noted that the weight loss value of NiO is negligible, meaning a whole dissolution of residual precursors and the high crystallinity degree of the as-prepared NiO sample. However, the TGA curve of 3DGH divides mainly into two stages: first stage, the weight loss in the temperature range of 25–200 °C is ascribed to the removal of the interlayered adsorbed water molecules; second stage, the eventual weight loss has been occurred after 200 °C is ascribed to the complete degradation and decomposition of the 3DGH sample. For the 3DGH/NiO25 nanocomposite, the initial weight loss is estimated to be 9.4 wt% owing to the removal of the interlayered adsorbed water molecules in the temperature range of 25–200 °C. The major weight loss beyond 450 °C can be attributed to the complete decomposition of 3DGH from the 3DGH/NiO25 nanocomposite. These results indicated that the as-synthesized NiO sample possesses a superior chemical stability, which is greatly, improved the thermal stability of the 3DGH/NiO25 nanocomposite.

**Fig. 3:**
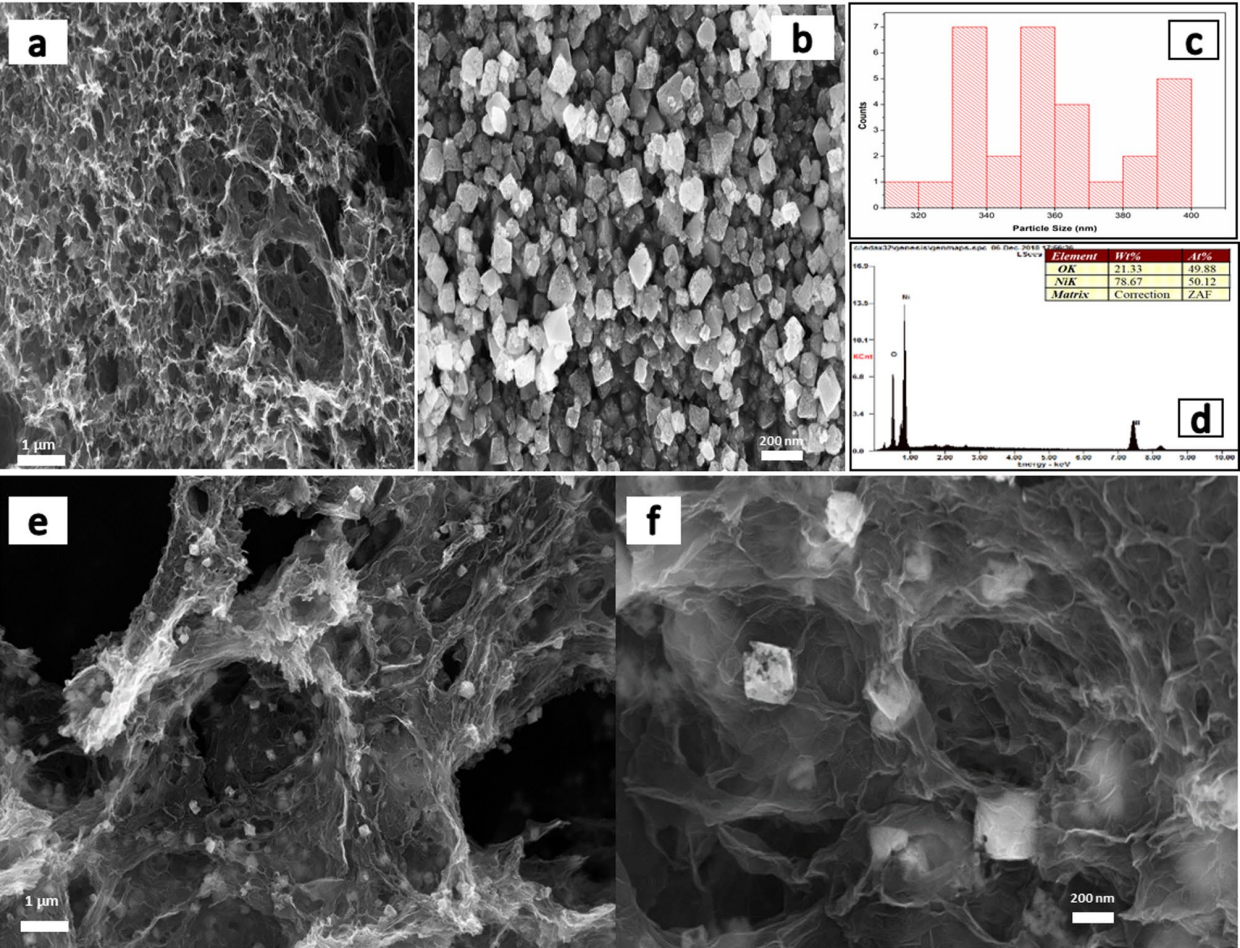
FE-SEM images of (**a**) 3DGH; (**b**) NiO octahedrons; (**c**) a histogram showing the particle size distribution of NiO octahedrons; (**d**) the EDS spectrum of the NiO octahedrons; and (**e**, **f**) low and high magnification of 3DGH/NiO25 nanocomposite.

The morphological characterization of 3DGH, NiO, and 3DGH/NiO25 has been clearly characterized by using FE-SEM and displayed in Fig. 3. The structure of 3DGH in Fig. 3a shows a three-dimensional (3D) architecture with the presence of mesopores and macropores. Figure 3b shows the FE-SEM image of nickel oxide nanostructure. It is clear that the obtained NiO displays octahedrons-like structure which is had a good uniform size with an average edge length varies from 300 to 400 nm. More importantly, there are a lot of nanopores on the surface of NiO octahedrons, which might be attributed to the etching process of silicon dioxide from as-synthesized NiO octahedrons products using a hot aqueous sodium hydroxide. Furthermore, the particle size distribution indicates that most particles fall within the 300–400 nm range (Fig. 3c). EDS analysis was conducted to determine the percent of elements in the NiO octahedrons As shown in Fig. 3d, the weight percent of Nickel (Ni) and Oxygen (O) are 78.67, and 21.33%, respectively. Figure 3e, f display the low and high magnification FE-SEM images of the 3DGH/NiO25. It can be clearly seen that the NiO octahedrons exhibits a well-decorated 3D graphene hydrogel and the graphene clearly embedded the NiO among its sheets and pores, forming a unique 3DGH/NiO25 hybrid nanostructure. It suggests that the nanopores on the surface of NiO octahedrons plays an important role in increasing the specific surface area and form stronger interfacial functionalization^48^. In addition, the FE-SEM images in Figure S1 of 3DGH/NiO5, 3DGH/NiO15 and 3DGH/NiO35, respectively, confirms the NiO octahedrons have been clearly decorated the 3DGH.

**Fig. 4:**
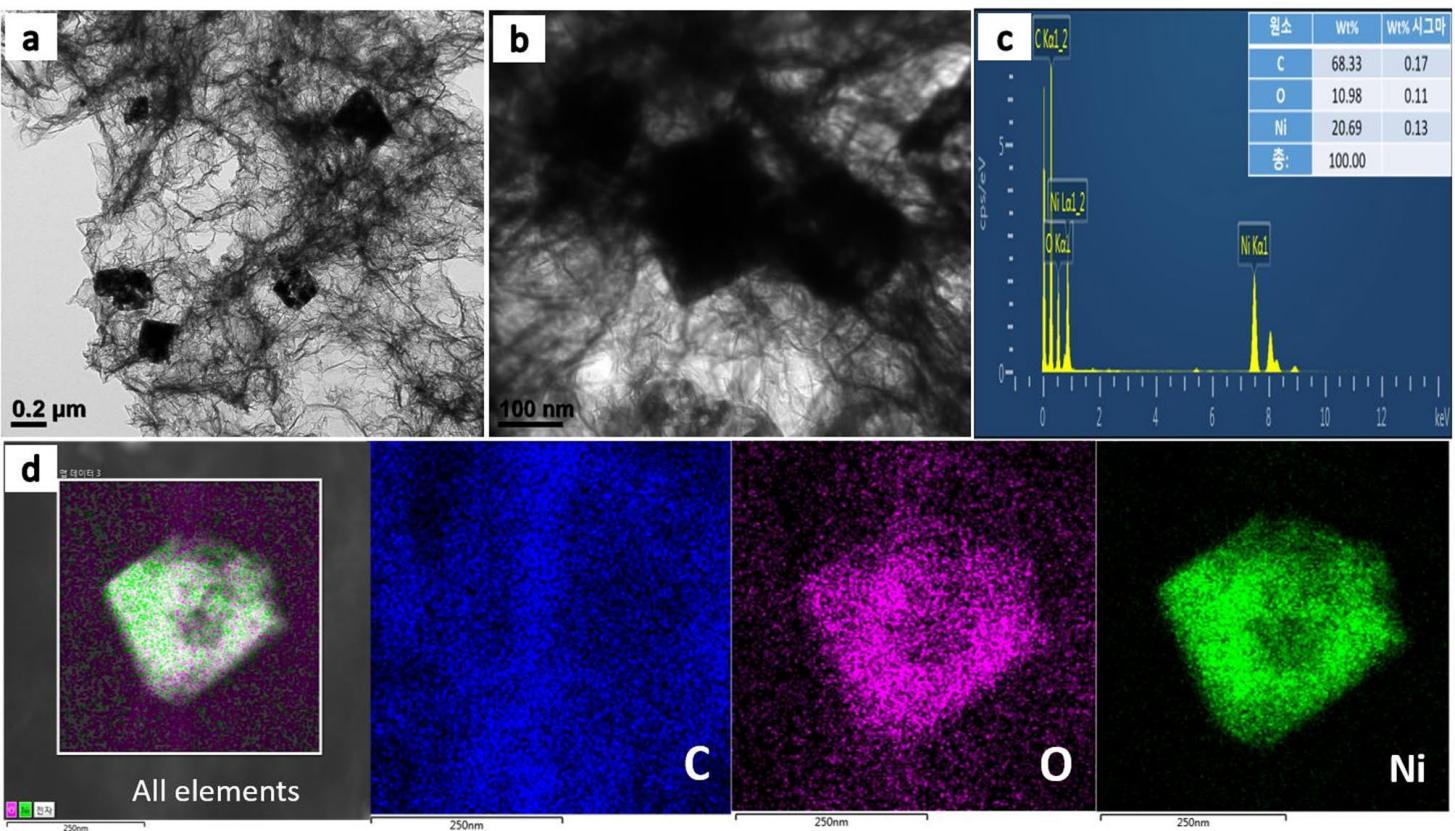
(**a**, **b**) Low and high magnification TEM images of the 3DGH/NiO; (**c**; **d**) TEM EDX analysis and TEM mapping of the 3DGH/NiO25 for the following elements: C, O, and Ni.

**Fig. 5:**
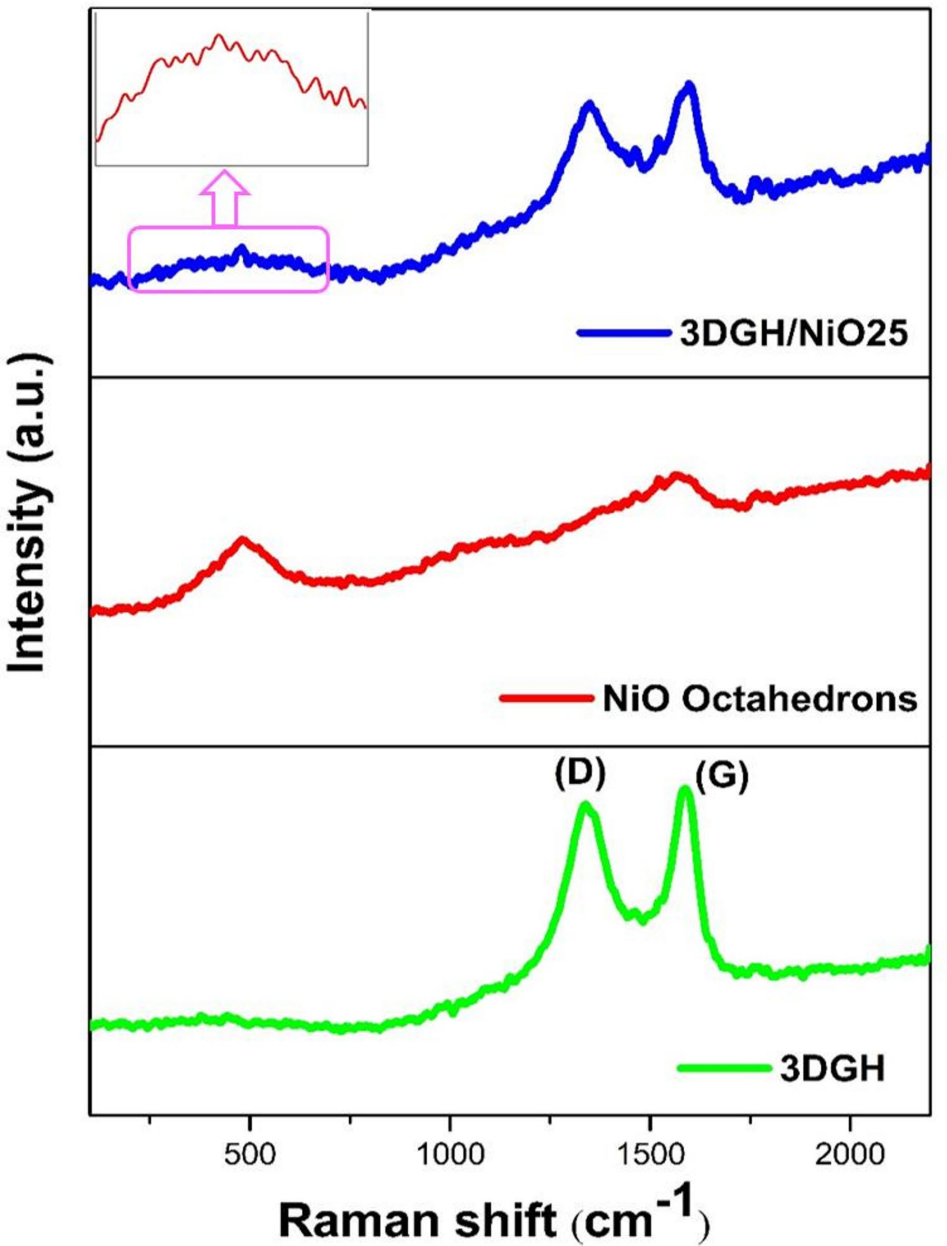
Raman spectra of the 3DGH, NiO octahedrons and 3DGH/NiO25.

Transmission electron microscope (TEM) technique was performed to depict the internal structure of the 3DGH/NiO25. As shown in Fig. 4a, it can be clearly noted that there are dark octahedrons spots on the 3D graphene hydrogel, indicating the NiO octahedrons samples were successfully maintained its morphology after self-assembly with the 3D graphene hydrogel. Figure 4b shows the TEM image at high magnification, it can be seen that an average edge length of the octahedrons is 300 nm and the NiO octahedrons seems to be encapsulated by 3D graphene hydrogel. Moreover, EDS analysis was conducted to determine the percent of elements in the 3DGH/NiO25 nanocomposite. As shown in Fig. 4c, the weight percent of Carbon (C), Oxygen (O) and Nickel (Ni) are 68.33, 10.98, and 20.69%, respectively. The presence and distribution of C, O, and Ni elements in the 3DGH/NiO25 nanocomposite were confirmed by STEM elemental mapping (Fig. 4d). The results of selected area showed that the mapped blue, purple, and green zones assigned to C, O, and Ni elements, respectively. On the other hand, the amount of oxygen was too much in the NiO octahedrons, whereas the 3D graphene hydrogel had lower oxygen content, which was in good agreement with XRD patterns. Furthermore, the crystallinity and microstructure of these materials was further analyzed by Raman spectroscopy analysis. As can be seen in Fig. 5, 3DGH and 3DGH/NiO25 nanocomposite clearly exhibits well-documented two characteristic peaks centered at 1349 cm^−1^ and 1582 cm^−1^ which were indicative of the D and G band, respectively. The first band (D) is related to the structural defects and disorders from the vibrations of sp^3^ carbon atoms and the next band (G) is related to the vibration of sp^2^ hybridized carbon atoms in the graphitic 2D hexagonal lattice ^49^.

Raman spectra of NiO octahedrons displays two obvious peaks, the first band at 500 cm^−1^ is assigned to the first-order longitudinal optical (LO), while the second band at 1090 cm^−1^ is due to 2LO phonon modes ^50,51^. In addition, a peak at 500 cm^−1^ is clearly observed in 3DGH/NiO25 Raman spectrum, which provides an evidence of the NiO octahedrons has been existed in the new nanocomposite. The peak ratio of the D and G band (I_D_/I_G_) has been testified to investigate the ordered and disordered the crystallized structures of carbonaceous materials ^52^. The intensity ratio (I_D_/I_G_) of the 3DGH/NiO25 (0.94) was significant higher than that in the 3DGH (0.91). This reveal that the defects and disordered graphite increases after 3DGH is decorated by NiO.

### Electrochemical measurements

#### Electrochemical impedance spectroscopy

**Fig. 6:**
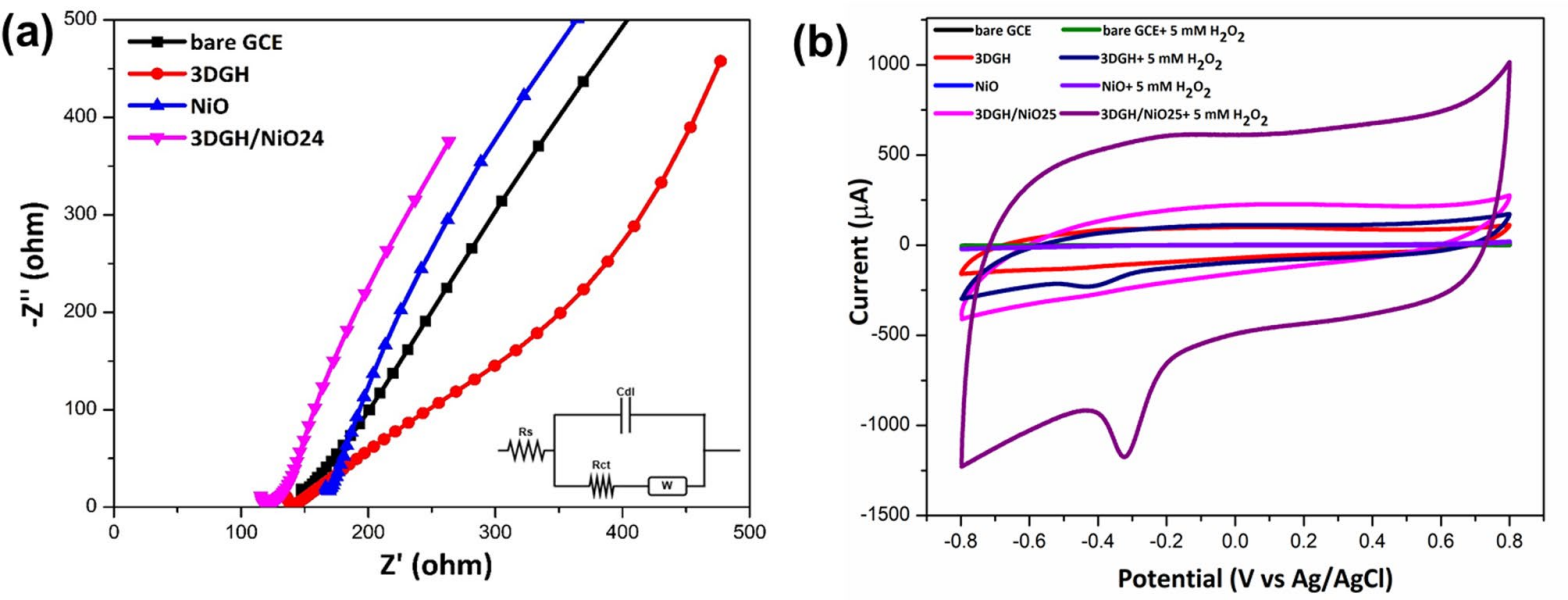
(**a**) Nyquist plots of bare, 3DGH, NiO and 3DGH/NiO25 electrodes in 5.0 mM K_3_Fe[CN]_6_ containing 0.1 M KCl from 0.1 Hz to 100 kHz at an AC amplitude of 5 mV. (**b**) CVs response curves of studied electrodes in the absence and presence of 5 mM H_2_O_2_ in 0.1 M PBS (pH-7.4) at 50 mV/s.

To ascertain the origin of the conductivity properties of as-prepared samples, we used an equivalent circuit to simulate the electrochemical impedance spectroscopy (EIS) in aqueous solution consisting of 5 mM [Fe(CN)_6_]^−3/−4^ and 0.1 M KCl as electrolyte with a frequency range from 1 Hz to 1 MHz at 5 mV amplitude. In the high frequency zone, a semicircle arc section of the Nyquist plots represents a diffusion resistance rate of electrolytes through electrode interface. The EIS spectra (Fig. 6a) were fitted using a Randles equivalent circuit. The extracted Rct values confirm the enhanced charge transfer properties of the 3DGH-modified electrodes. Notably, the 3DGH/GCE and 3DGH/NiO25/GCE electrodes showed the lowest Rct values, reflecting their superior electron transfer kinetics and catalytic efficiency toward H₂O₂ reduction. As shown in Fig. 6a, it is clear that the 3DGH/NiO25 electrode exhibits the smallest charge transfer resistance (R_ct_ ~ 98 Ω) as compared to the bare GC electrode (R_ct_ ~ 140 Ω), 3DGH electrode (R_ct_ ~ 129 Ω) and NiO electrode (R_ct_ ~ 152 Ω), revealing the superior electron transfer process of the fabricated 3DGH/NiO25 electrode. Moreover, the Wargburg resistance (W_d_) reflects the capacitive behavior of the developed electrodes at low frequency zone via the straight line section^53–55^.

Obviously, the straight line of the 3DGH/NiO25 electrode are much closer to the Y axis compared to the other prepared electrodes, suggesting that the 3DGH/NiO25 electrode has very small ion diffusion resistance with the ideal capacitive behavior^56^. That means the 3DGH/NiO25 nanocomposite has unique conductivity features for the electrochemical biosensing application.

#### Electrocatalytic properties toward H_2_O_2_ reduction

In order to evaluate the electrocatalytic activity of as-fabricated electrodes on the detection of H_2_O_2_, cyclic voltammetry (CV) were conducted in N_2_-saturated 0.1 M PBS (pH = 7.4) in the absence and presence of 5 mM H_2_O_2_ at a scan rate of 50 mV s^−1^ using a conventional three-electrode system including Ag/AgCl and a platinum wire as the reference and the counter electrodes, respectively. As shown in Fig. 6b, there were no peaks on all working prepared electrodes in 0.1 M PBS for the absence of H_2_O_2_. After injecting 5 mM H_2_O_2_ into 0.1 M PBS as shown in Fig. 6b, although the bare GCE shows a minor cathodic wave at − 0.35 V upon H₂O₂ addition, the response is minimal and lacks a well-defined peak. This indicates poor electrocatalytic activity, in contrast to the strong and sharp reduction observed with the 3DGH/NiO25/GCE. Among four 3DGH/NiO nanocomposites, the 3DGH/NiO25 shows the highest current reduction response than other composites (Figure S3). The superior electrochemical catalytic activity of 3DGH/NiO25/GCE towards H_2_O_2_ reduction could be ascribed to the good synergistic between 3DGH and NiO octahedrons which increased the electron transfer rate and led to further improve the detection process of H_2_O_2_. Furthermore, the significant enhancement in cathodic current response observed for 3DGH/NiO25/GCE, compared to other electrodes at the same potential, indicates a more favorable electrocatalytic environment for H₂O₂ reduction. The electrochemical reduction of H_2_O_2_ on the 3DGH/NiO25/GCE surface likely follows a two-electron transfer pathway as Eq. (1):

1 $${H}_{2}{O}_{2}+{2e}^{-}\to 2{OH}^{-}$$

**Fig. 7:**
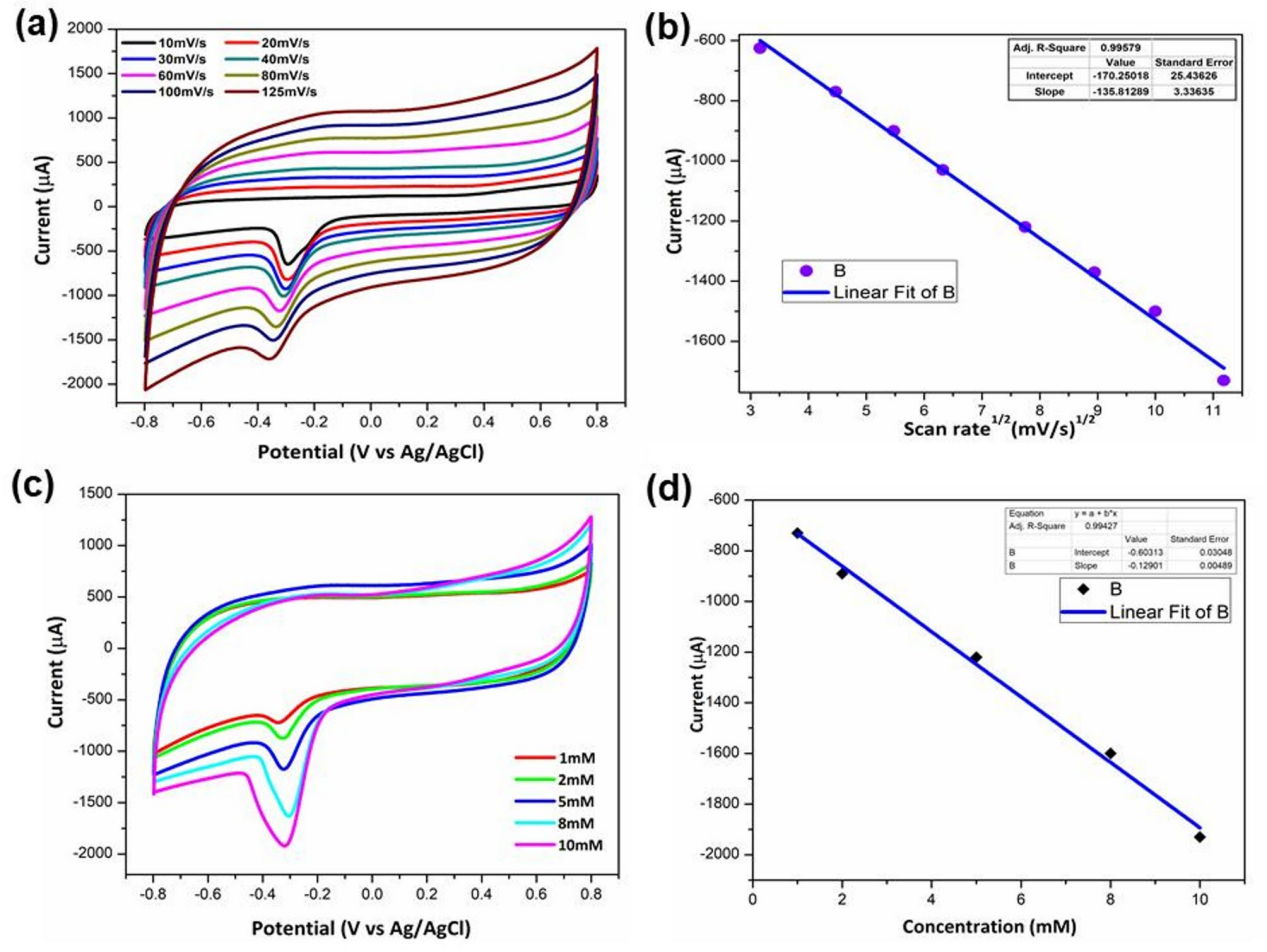
(**a**) CV curves of the 3DGH/NiO25/GCE electrode at different scan rates in N_2_ saturated 0.1 M PBS solution containing 5 mM H_2_O_2_. (**b**) Plot of reduction peaks current vs. square root of scan rates. (**c**) CV curves of the 3DGH/NiO25/GCE electrode in 0.1 M PBS with different concentrations of H_2_O_2_ at a scan rate of 50 mV s^−1^. (**d**) Plot of reduction peaks current vs. concentrations of H_2_O_2_.

The 3D graphene hydrogel provides a high surface area and conductive network that enhances electron mobility and promotes efficient contact between H₂O₂ molecules and the electrode surface^57^. Additionally, NiO nanoparticles act as active catalytic centers, facilitating electron transfer by undergoing redox transitions^58^. The reduction of H₂O₂ on the 3DGH/NiO25/GCE is proposed to follow a two-electron pathway forming OH⁻, consistent with previously reported mechanisms on NiO-based^58^ and graphene-based electrodes^57^, revealing the synergistic interaction between NiO and 3DGH further enhances the catalytic behavior. The electrochemically active surface area was evaluated by estimating the double-layer capacitance in the non-faradaic region via cyclic voltammetry. The 3DGH/NiO25/GCE exhibited of 44.7 μF/cm^2^, significantly higher than that of the bare GCE (11.9 μF/cm^2^), indicating an increased electrochemical surface area. The increased current observed in the presence of H₂O₂ is attributed mainly to faradaic processes rather than purely capacitive behavior. The synergistic integration of NiO with 3DGH facilitates improved charge transfer and enhanced accessibility of active sites, thereby accelerating the H₂O₂ electro-reduction process^59–61^. To further demonstrate excellent electrocatalytic activity of 3DGH/NiO25 nanocomposite for reduction of H_2_O_2_, cyclic voltammetry (CV) was carried out in 0.1 M PBS with 5 mM H_2_O_2_ at different scan rates ranging from 10 to 125 mV s^−1^. As can be seen from Fig. 7a, the cathodic peak currents which represent the reduction process of H_2_O_2_ on the surface of optimized electrode increased gradually with the increase in scan rate. Furthermore, the cathodic peak potential exhibits a gradual shift toward more negative values. This behavior is characteristic of an irreversible or quasi-reversible electrochemical process, where the electron transfer kinetics are not fast enough to keep up with the increasing scan rate^62^. This shift is typically attributed to the higher overpotential required to drive the redox process at faster scan rates due to limitations in electron transfer and diffusion rates. Similar trends have been observed in previous studies for non-enzymatic H₂O₂ sensors^63–66^. Besides that, the cathodic peak currents were found to increase linearly with the square root of the scan rate (Fig. 7b), which indicates a surface diffusion controlled process^67^. Additionally, the catalytic activity of 3DGH/NiO25/GCE towards H_2_O_2_ was investigated at 50 mV s^−1^ using different concentration of H_2_O_2_. As seen from Fig. 7c, the cathodic peak current increased with increasing H_2_O_2_ concentrations and a good linear relationship was found between the current reduction peak and the concentration of H_2_O_2_ (Fig. 7d), demonstrating the excellent electrocatalytic activity of 3DGH/NiO25 nanocomposite towards H_2_O_2_ reduction.

#### Amperometric H_2_O_2_ detection on the 3DGH/NiO25/GCE

**Fig. 8:**
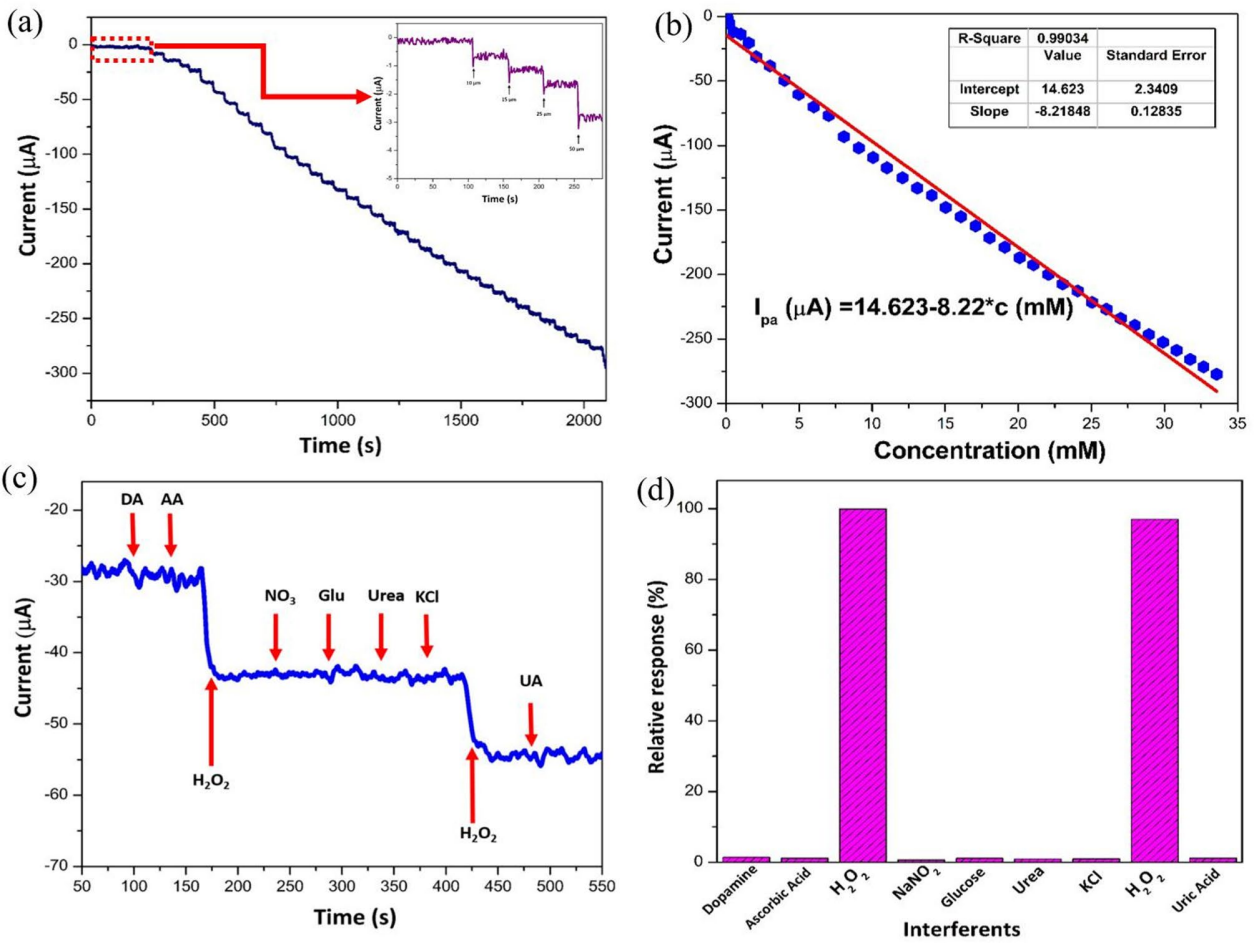
(**a**) Amperometric response of 3DGH/NiO25/GCE on successive additions of H_2_O_2_ into 0.1 M PBS at − 0.35 V. Inset shows a blown-up image of the low-concentration region. (**b**) Calibration curve between H_2_O_2_ concentration and amperometric response. (**c**) Interference test of the 3DGH/NiO25/GCE/GCE electrode in 0.1 M PBS at − 0.35 V with 0.5 mM H_2_O_2_ and other interferents including 1 mM of DA, AA, NaNO_2_, Glucose, Urea, KCl and UA. (**d**) The corresponding bar diagram of the selectivity to different interfering species.

Amperometric technique at a fixed potential further corroborates the electrocatalytic activities of electrochemical sensors. Figure 8a exhibited the amperometric current–time responses of the 3DGH/NiO25 with successive step changes of H_2_O_2_ concentrations into 0.1 M PBS (pH 7.4) with the solution stirred constantly through static applied potential at − 0.35 V. It can be clearly observed that the current response of the sensor changed rapidly after each addition of H_2_O_2_ and reached a steady state in approximately 7 s, indicating a fast electrocatalytic response of our fabricated sensor to H_2_O_2_ detection.

The calibration curve of 3DGH/NiO25 on consecutive addition of various concentrations of H_2_O_2_ with time interval around 50 s is shown in Fig. 8b. The as-fabricated electrode displayed a wide linear detection range from 10 µM to 33.58 mM and the linear regression equation can be described as ${I}_{pa}\left(\mu A\right)=14.623-8.22c (mM)$ with a correlation coefficient of 0.9903. The sensitivity is calculated to be 117.26 µA mM^−1^ cm^−2^ and a detection limit of 5.3 µM at a signal-to-noise ratio of 3. As observed in Table 1, the performance sensing of the proposed sensor is comparable and even superior to other previously reported H_2_O_2_ sensors with regard to graphene/metal oxides nanocomposites. This may attribute to the admirable properties of the NiO octahedrons, which played the significant part in demonstrating satisfactory detection result with a wide linear detection range and lower limit of detection.

**Table 1:** Comparison of the performance of the as-fabricated 3DGH/NiO25 sensor with the other reported H_2_O_2_ sensors.

Electrode	Linear range (µM)	Sensitivity (μA mM^−1^ cm^−2^)	Detection limit (µM)	Refs.
NiO/graphene	250–4750	591	0.7667	^68^
NiO-MNS	10–800	236.7	0.62	^69^
NiO/α-Fe_2_O_3_	500–3000	146.98	50	^70^
NiO-NSs/CF-1801/GCE	200–3750	23.3	0.01303	^71^
NiO-MoS_2_	5–455	3925	3	^72^
NiO-PE(A4)	Up to 4000	25	5	^73^
NiO-MNS	0.01–0.8	236.67	0.62	^69^
Fe_3_O_4_/graphene	0.8–334.4	274.15	0.078	^74^
Cu_2_O/N-graphene	5–3570	26.67 μA mM^−1^	0.8	^75^
Au-TiO_2_/graphene	10–200	151.5	0.7	^76^
N-Co-CNT@graphene	2–7449	28.66	2.0	^77^
PDDA/graphene	0.5–500	140.8	0.1	
Cu_2_O/graphene	300–7800	–	20.8	^2^
3DGH/NiO25	10–33,580	117.26	5.3	This Work

#### Selectivity, reproducibility, stability and real sample analysis

**Fig. 9:**
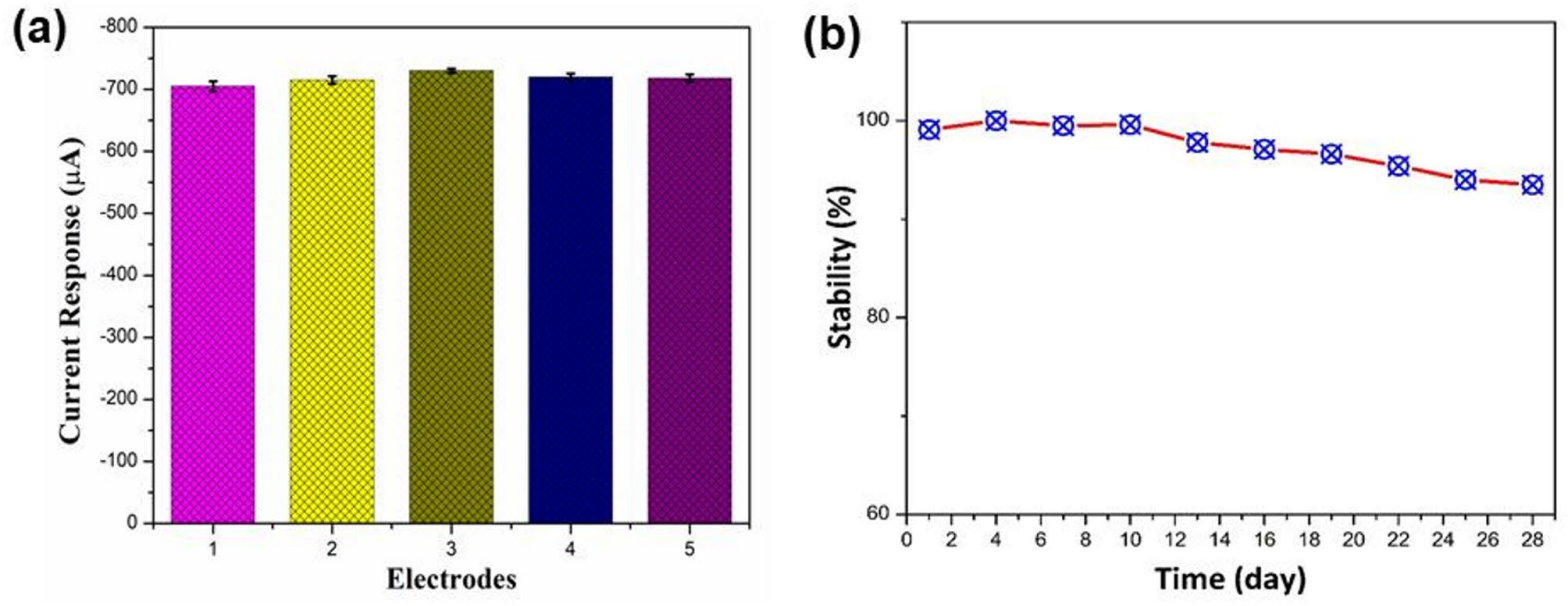
Reproducibility study for five electrodes of 3DGH/NiO25/GCE; (**b**) Stability test of the 3DGH/NiO25/GCE for one month.

Furthermore, the selectivity test of electrochemical sensors has become a critical area of focus, extensively employed to verify a sensor’s ability to accurately detect target analytes amid potential interferences. To assess the selectivity test of the 3DGH/NiO25 nanocomposite modified electrode, the amperometric measurements were conducted at applied potential of − 0.35 V (vs. Ag/AgCl) in 0.1 M PBS (pH 7.4) solution with initial addition of 1 mM H_2_O_2_ and then 2 mM of each interfering species i.e. dopamine (DA), ascorbic acid (AA), NaNO_2_, glucose, urea, KCl and uric acid (UA). As clearly shown in Fig. 8c and d, a rapid current signal response was obtained with the addition of 1 mM H_2_O_2_. Nevertheless, no remarkable current signal change was observed upon addition of interfering species, which demonstrates the higher selectivity of the 3DGH/NiO25 developed sensor toward H_2_O_2_ molecules compare to the interfering species. Moreover, the reproducibility of this proposed electrochemical sensor was investigated by measuring the current signal response of five similarly modified electrodes in 0.1 M PBS (pH 7.4) containing 1 mM H_2_O_2_. The results shown on Fig. 9a revealed that the current signal response of all modified electrodes were obtained almost same values at 1 mM H_2_O_2_, with relative standard deviation (RSD) of 4.3% Additionally, the long-term stability was regarded as one of the most significant factors to monitor the performance of the proposed sensor. To evaluate the long-term stability of the modified electrode, the current signal response of reduction peak was measured via cyclic voltammetry every three days for one month. As can be seen in Fig. 9b, the response of modified electrode decreased to 93.5% of its initial value after being stored for one month under ambient conditions. Therefore, the superior analytical results including selectivity, reproducibility and long-term stability of our fabricated sensing electrode based on 3DGH/NiO25 indicate that practically viable of sensor to detect H_2_O_2_ in a real study. Therefore, the analytical utilities of the 3DGH/NiO25 proposed electrode were conducted to detect H_2_O_2_ in real products of commercial milk samples.

For sample preparation, one hundreds microliter of commercial milk was diluted into 20 ml of 0.1 M PBS (pH 7.4) solution and then analyzed by amperometric technique at − 0.35 V with an appropriate known amounts of H_2_O_2_. The recovery and the accuracy values of the as-fabricated electrode were achieved by the standard addition method. The 3DGH/NiO25 nonenzymatic sensor exhibits high values of recovery percentage with the relative standard deviations (RSD%) of lower than 3.32%, as presented in Table S1. These satisfactory results further proved that the as-fabricated 3DGH/NiO25 sensing electrode is a feasible promising candidate for the detection of H_2_O_2_ in the environmental life system.

## Conclusions

In this study, a novel approach was utilized for synthesizing NiO octahedrons. In addition, the self-assembly of NiO octahedrons decorated 3DGH via a hydrothermal method and applied for the electrochemical detection of H_2_O_2_. Compared with pure 3DGH, NiO octahedrons and four nanostructures of 3DGH/NiO, the 3DGH/NiO25 nanostructure exhibited superior sensing material toward H_2_O_2_ detection, including high sensitivity (117.26 µA mM^−1^ cm^−2^) with wide linear detection range (10 µM-33.58 mM) and low detection limit (5.3 µM) at a signal-to-noise of 3. Therefore, the 3DGH/NiO25 nanocomposite with superior performance has practical values for H_2_O_2_ detection in real samples.

## Electronic supplementary material

Below is the link to the electronic supplementary material.

Supplementary Material 1

## Data Availability

The datasets used and/or analyzed during the current study are available from the corresponding author on reasonable request.
