# Supplementary material for: Enzymeless electrochemical detection of hydrogen peroxide using NiO octahedron decorated 3D graphene hydrogel
Source: Sci Rep. 2025 Aug 3;15:28293. doi: 10.1038/s41598-025-10472-6 (PMC12319076; doi:10.1038/s41598-025-10472-6)
Supplement: Supplementary file 1 — Supplementary Material 1 [file 41598_2025_10472_MOESM1_ESM.docx]

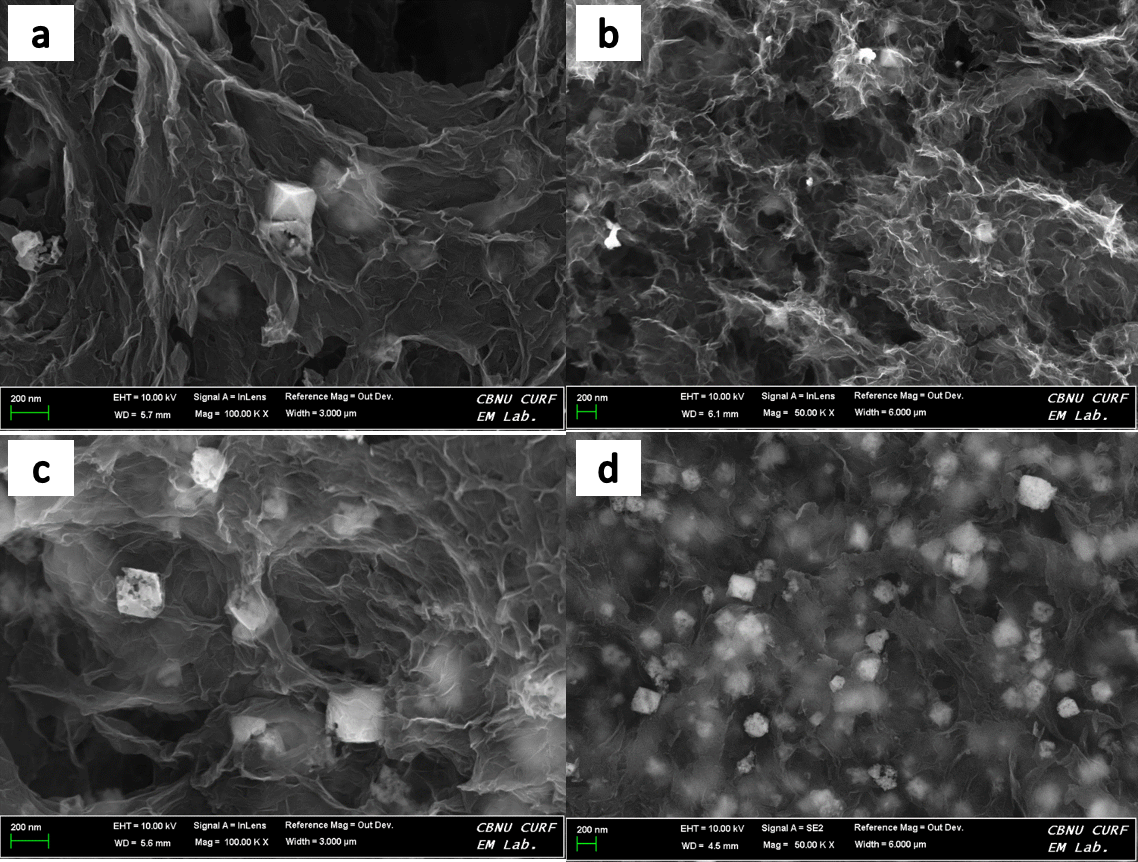


Figure S1. FE-SEM images of 3DGH/NiO5 (a), 3DGH/NiO15 (b), 3DGH/NiO25 (c) and 3DGH/NiO35 (d).


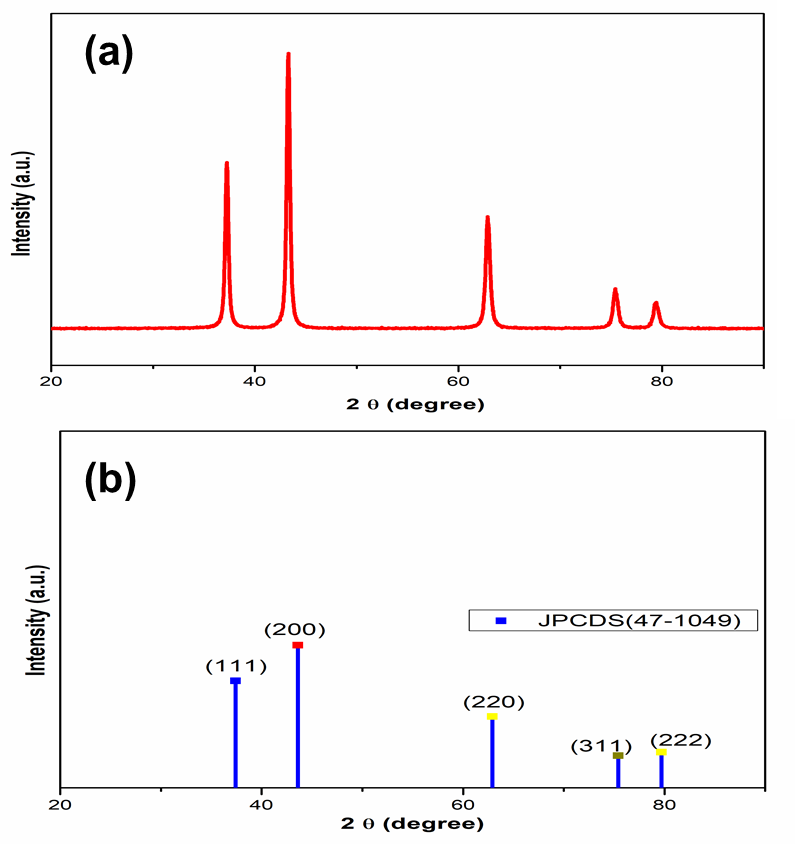


Figure S2. XRD pattern of NiO nanoparticles overlaid with the standard reference peaks (JCPDS Card No. 47-1049).

Table S1. Crystallite size estimation of NiO before and after composite formation (3DGH/NiO) using the Debye–Scherrer equation.

| **NiO Sample** | **Diffraction Peak** | **2 θ degree** | **FWHM** | **Crystallite size (nm)** |
| --- | --- | --- | --- | --- |
| **Before** | **200** | **43.3** | **0.323^0^** | **26.5** |
| **After** | **200** | **43.3** | **0.359^0^** | **23.8** |


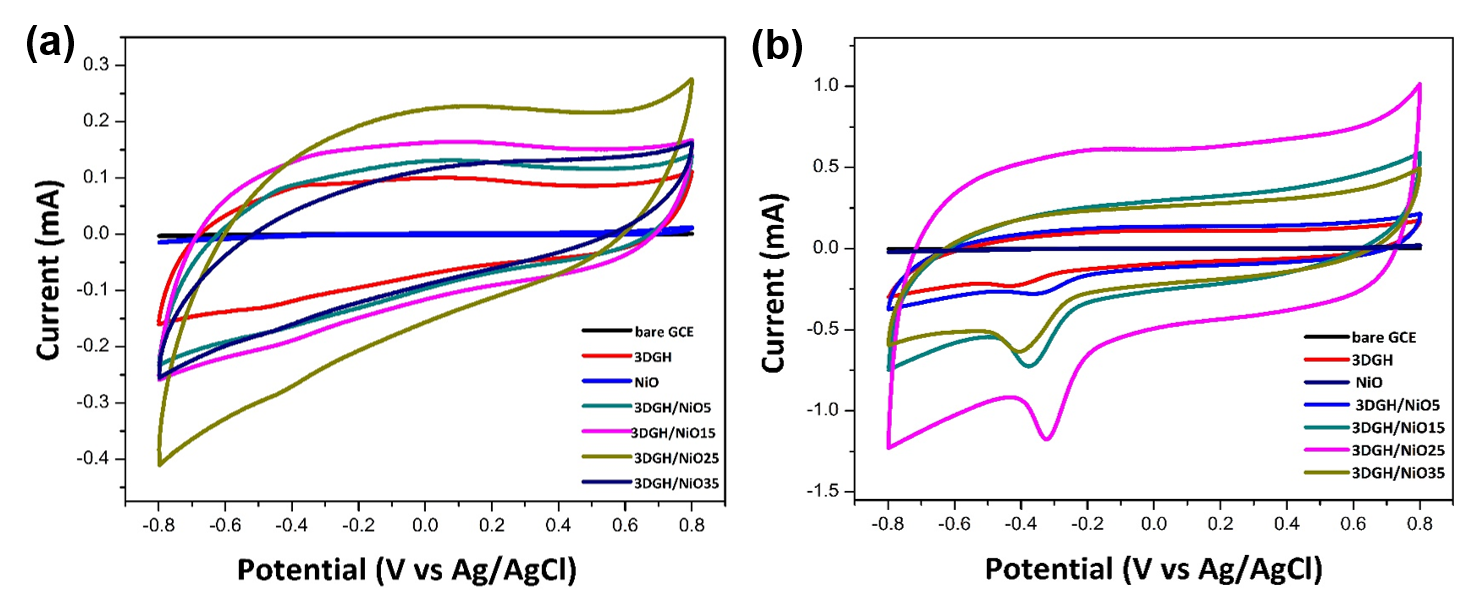


Figure S3. CVs response curves of studied electrodes in the (a) absence and (b) presence of 5 mM H_2_O_2_ in 0.1 M PBS (pH-7.4) at 50 mV/s.

Table S1. Determination of H_2_O_2_ in commercial milk samples using 3DGH/NiO25/GCE.

| **Sample** | **Added (mM)** | **Found (mM)** | **Recovery (%)** | **RSD (%)** |
| --- | --- | --- | --- | --- |
| 1 | 0.2 | 0.184 | 92 | 3.32 |
| 2 | 0.4 | 0.748 | 93.5 | 2.87 |
| 3 | 1.5 | 1.434 | 95.6 | 3.19 |
